# Eating Behaviors in Early Childhood (1-5 Years) and Their Association With Sociodemographic Characteristics in Turkey

**DOI:** 10.7759/cureus.16876

**Published:** 2021-08-04

**Authors:** Cengiz Gökalp, Süleyman Yıldız, Oya Halıcıoğlu Baltalı

**Affiliations:** 1 Pediatrics and Child Health, Mardin Derik State Hospital, Mardin, TUR; 2 Pediatrics and Child Health, University of Health Sciences İzmir Tepecik Training and Research Hospital, İzmir, TUR

**Keywords:** eating behavior, early childhood, bottle feeding, appetite, eating habits

## Abstract

Eating problems are common in childhood and being seen in 25%-45% of healthy children. The period following the first year of life up to five years is when eating problems are most frequently reported and also when the child acquires eating habits. Several studies have shown that eating behavior is affected by the family environment, and by parental eating habits and modes of feeding their children.

The purpose of this study was to investigate the eating behavior characteristics of children in early childhood, to determine the frequency of problematic eating behaviors, and to compare these with the family’s sociodemographic characteristics.

The study consisted of 255 children aged 1-5 years, with no chronic disease, presenting to İzmir Tepecik Education and Research Hospital Child Health and Diseases Department general pediatric clinics between February and April, 2017, together with their parents. In the study, 39 questions were asked to parents related to children’s demographic characteristics and eating behaviour.

Problematic eating behavior was present in 43.9% of the study group. Analysis identified feeding bottle use, feeding with the use of an assistant object, eating lasting longer than half an hour, and the child not feeding itself as the most frequent eating problems. Malnutrition was present in 104 (65.8%) of the children with problematic eating behavior. Examination of unhealthy eating behavior in the light of the study findings showed no significant association between eating behaviors and variables such as type and time of delivery, maternal education level, maternal employment status, maternal age, or the number of children in the family.

In terms of nutrition status, Turkey appears to exhibit problems associated with both developed and developing countries. Public nutrition in Turkey fluctuates significantly depending on the region, the season, socioeconomic levels, and differences between urban and rural settlements. Early-onset of eating-related problems can have a negative effect on children’s subsequent physical, emotional, and social development.

Determination in the early period of eating problems seen in children and investigation of the causes thereof is important in terms of identifying a potential solution. It is important to work with the family to that end, and for children to be followed-up by pediatricians at regular intervals.

## Introduction

Eating is a complex behavior associated with both biological and social factors [1]. Eating problems are common in children and occur in 25%-45% of healthy children, and in ≥80% of children with developmental delay [2]. Studies on healthy children show that 20%-60% of parents think their children do not eat sufficiently [3,4]. Severe eating disorders requiring intensive behavioral therapy occur in 3%-10% of all children [4,5].

Eating problems are most frequently reported in children aged 1-5 years, which is also the period during which children acquire eating habits. Early-onset eating-related problems can have a negative effect on subsequent physical, emotional, and social development [5]. The development of eating behaviors in childhood is a complex process and eating disorders can develop at any time during childhood. The family environment is where children have their first experiences with eating and the introduction of foods. Several studies have shown that eating behavior is affected by the family environment, and by parental eating habits and modes of feeding their children [1,2].

The present study aimed to determine the eating behavior characteristics of young children, to determine the frequency of problematic eating behaviors, and to determine the association between the children’s eating behaviors and family sociodemographic characteristics.

## Materials and methods

The study included 255 children without chronic disease aged 1-5 years (and their parents) presented to İzmir Tepecik Education and Research Hospital, Child Health and Diseases Department, İzmir, Turkey, between February 2017 and April 2017. Parents were sent a questionnaire consisting of 39 items to collect data on sociodemographic characteristics of the child and the family, child anthropometric characteristics, such as body weight, height, and weight for height (WFH), and child eating behaviors. The study protocol was approved by the Health Sciences University İzmir Tepecik Education and Research Hospital Ethics Committee, Izmir, Turkey (decision no. 17; 26.01.2017). 

The first 16 questionnaire items were related to sociodemographics. Questions 19, 21, and 24-33 (12 items in total) evaluated children’s eating behaviors. Each positive eating behavior was scored 0, whereas each negative eating behavior was scored 1. The highest possible score was 12, with higher scores indicating poorer eating behavior. A median total eating behavior score was calculated, with scores above the median value indicating problematic eating. A mean score was then calculated for the eating behavior items, and comparisons between groups were performed using parametric methods. Questions 17 and 18 were about the total duration of breastfeeding and the duration of exclusive breastfeeding. Other items related to eating were item 20 (the time supplementary feeding started), item 22 (mother’s perception of her child’s appetite), item 23 (the perception of whether or not an eating problem existed, and item 34 (whether other individuals in the family had eating-related problems, such as lack of appetite, selective eating, or irregular eating).

Mothers were grouped based on their level of education; those with a middle school or lower level constituted the low education group, and those with high school and above level of education constituted the well-educated group. The groups were then compared in terms of the children’s eating behavior scores. In 2017, the minimum wage was announced as 1400 TL by the government. In Turkey, below minimum wage is accepting as the poorest level of income people have. Therefore, those with an income of less than 2000 TL were evaluated as the low-income group and compared their eating scores. 

The children were evaluated in terms of WFH; those with a WFH <90% were considered to have protein-energy malnutrition, and those with a WFH >90% were considered not to have a protein-energy deficiency. The 2 WFH groups were then compared in terms of eating behavior scores. Children with a median eating behavior score indicating an eating disorder were then compared with the other children regarding the presence or absence of protein-energy malnutrition.

Data analysis

Statistical analysis was performed using IBM SPSS Statistics for Windows v.20.0 (IBM Corp., Armonk, NY, USA). Numerical data are shown as mean ± SD, and categorical data are shown as percentage and frequency. The chi-square test was used to determine the relationships between demographic data and eating behaviors. The level of statistical significance was set at P < 0.05.

## Results

The mean eating behavior score of the entire study group was 5.31 ± 2.06 (range: 0-11), and the median score was 5. Children with a median score ≥5 were considered to have problematic eating behavior. Accordingly, 112 (43.9%) of the children exhibited such behavior.

Findings associated with demographic characteristics

In total, 140 (54.9%) of the 255 children were male, and 115 (45.1%) were female. Among the children, 90 (35.3%) were born via the normal spontaneous vaginal route and 165 (64.7%) via cesarean delivery. In all, 200 of the children were born full-term, and 55 were born premature. The mean eating behavior score was 5.29 ± 2.11 in children born full-term, versus 5.41 ± 1.91 in those born prematurely; the difference was not significant (P = 0.672).

In terms of family demographic data, the age of the children in the study group was homogeneous, ranging between 1 and 5 years. 80.8% (n = 206) of the caregivers were mothers, 0.4% (n = 1) were fathers, 13.7% (n = 35) were grandmothers, 2% (n = 5) were professional caregivers, and 3.1% (n = 8) were other individuals. Among the mothers, 53 (20.8%) worked, whereas 202 (79.2%) did not. The maternal level of education was generally low, as shown in Table 1. 

**Table 1:** Age distributions and birth weights of children, mothers’ education levels, and family income levels.

Variables	
	Mean ± SD
Age (months)	36.67 ± 1.65
Birth weight	2907.89 ± 778.35
Mothers' education status	Case number (%)
Uneducated	9 (3,5%)
Elementary school	79 (31.0 %)
Middle school	61 (23.9 %)
High school	64 (25.1 %)
University	42 (16.5 %)
Total	255 (100 %)
Family income level ( TL: Turkish lira)	
Below minimum wage	17 (6.7 %)
Minimum wage	111 (43.5 %)
Minimum wage -2000 TL	51 (20.0 %)
Above 2000 TL	76 (29.8 %)
Total	255 (100.0 %)

The mean eating behavior score in the children whose mothers were in the well-educated group was 5.20 ± 2.11, versus 5.39 ± 2.00 in those whose mothers were in the low education group; the difference was not significant (P = 0.481). The mean eating behavior score in the children whose mothers worked was 5.67 ± 2.26, as compared to 5.22 ± 2.00 in those whose mothers did not work; the difference was not significant (P = 0.187). There weren’t any significant differences between the working and non-working mothers in terms of the duration of breastfeeding (11.18 ± 7.61 months and 13.19 ± 8.23 months, respectively) or time of exclusive breastfeeding (5.49 ± 2.70 months and 5.43 ± 2.93 months, respectively) (P = 0.970 and P = 0.902, respectively).

Investigation of family monthly income showed that families were generally in the low-income group, as shown in Table 1.The mean eating behavior score in the children whose families had a monthly income <2000 TL was 5.38 ± 2.04 compared to 5.25 ± 2.09 in those with a monthly family income >2000 TL, and the difference was not significant (P = 0.621).

Findings associated with eating behavior

Data concerning problematic eating behavior was reported by parents (questionnaire items 19, 21, and 24-33; 12 questions in total). Individual question analysis and total scores are shown in Table 2.

**Table 2:** Problematic eating behaviors. *Favorable eating behavior; **unfavorable eating behavior.

	Yes (n, %)	No (n, %)
Use of baby feeding bottle? (item 19)**	205/80.4	50/19.6
History of difficulty swallowing solid foods? (item 21)**	42/16.5	213/83.5
Does the child feed himself? (item 24)*	156/61.2	99/38.8
Does the child sit down at the table with the family? (item 25)*	235/92.2	20/7.8
Are meals regular? (item 26)*	218/85.5	37/14.5
Does eating last less than half an hour? (item 27)*	127/49.8	128/50.2
Is any compulsion or intimidation applied to eat? (item 28)**	57/22.4	98/77.6
Is the child made to eat through TV-games-attention distracting objects? (item 29)**	136/50.3	119/40.7
Is the child made to eat by standing behind him? (item 30)**	81/22.4	174/68.6
Does the child keep food in its mouth for a long time? (item 31)**	83/32.5	132/67.5
Is the child a fussy eater? (item 32)**	139/54.5	116/45.5
Does the child consume snack foods? (item 33)**	155/60.8	100/39.2

In terms of responses to questions concerning eating other than the problematic eating section, responses to item 22, in which mothers evaluated their children’s appetites, are shown in Table 3.

**Table 3:** Mothers’ perceptions of their children’s appetites.

	Case number (%)
Poor appetite	72 (28.2 %)
Moderate	46 (18.0 %)
Good	128 (50.2 %)
Very good	9 (3.5 %)
Total	255 (100.0 %)

The mean eating behavior score was 6.20 ± 1.95 in the children described by their mothers as having a poor or moderate appetite, versus 4.55 ± 1.85 in those reported to have a good or very good appetite (Table 5) (P < 0.001). In all, 98 mothers (38.4%) responded positively, and 157 (61.6%) responded negatively to questionnaire item 23 (does your child have an eating problem). Analysis of questions concerning eating problems in the family (items 34, 35, and 36) showed that based on responses to item 34, 90 (35.3%) mothers reported eating problems in the family, whereas 164 (64.3%) did not. The family members with an eating problem distribution of types of eating problems in the families are shown in Table 4. 

**Table 4:** Distribution of eating problems and types among individuals in the family (n = 100).

Family member	Case number (%)
Mother	21 (21%)
Father	29 (29%)
Sibling	50 (50%)
Total	100 (100%)
Types of eating problems	
Fussy eating	61 (61%)
Lack of appetite, under-eating	32 (32%)
Skipping meals, irregular eating	5 (5%)
Overeating, excessive appetite	2 (2%)
Total	100 (100%)

Self-feeding in children is expected up to two years of age. The situation of feeding them by themselves or someone else is examined according to age groups and is shown in Table 5. 

**Table 5:** Self-feeding status by age groups.

Age group	1-2 years	Over 2
Self-feeding	n = 49, 43.8%	n = 107, 74.8%
Fed by others	n = 63, 56.2%	n = 36, 25.2%
Total	n = 112, 100%	n = 143, 100%

WFH analysis showed that 79 (31%) of the children had protein-energy malnutrition and 176 (69%) did not. There was a significant difference in the eating behavior score between the children with and without protein-energy malnutrition, as expected. The eating score of malnutrition positive children score was 5.75 ± 2.10, and malnutrition negative children score was 5.1200 ± 2.02 (P-value:0.025).

## Discussion

The period following the first year of life up to five years is when eating problems are most frequently reported and when the child acquires eating habits. The onset of problems in eating problems in the early period may adversely affect children's physical, emotional, and social development in the future [1,3]. Several studies report that 25% to 45% of developmentally healthy children and 80% of children with motor-mental developmental delay have eating problems. Problems in initiating solid foods (holding in the mouth, retching, vomiting, refusal to eat, etc.), feeding by parents who do not allow the child to eat himself, feeding by using objects, distracting games and walking behind the child, keeping the eating time long and compulsive eating behaviors are common eating problems [1,5].

Problematic eating behavior was noted in 43.9% of the present study group. Analysis showed that bottle feeding, feeding with the use of an attention-distracting object, eating lasting >30 min, and a child not feeding itself were the most frequent eating problems. Protein-energy malnutrition was present in 104 (65.8%) of the children with problematic eating behavior. According to the 2013 Turkey Demographic and Health Survey [6]; the prevalence of malnutrition in the general population (-2 SDS or <90%) is approximately 2%; the equivalent rate in the present study was 30%. We attribute this to the study being performed at the pediatric clinic of a tertiary hospital and that cases of growth development retardation and feeding problems were followed up alongside healthy children.

In terms of nutrition status, Turkey appears to exhibit problems associated with both developed and developing countries. Nutrition in Turkey fluctuates significantly according to region, season, socioeconomic level, and differences between urban and rural residences, which affects the type and prevalence of eating problems [6,7]. In the present study, there wasn’t a significant difference in eating behaviors between the children with a monthly income <2000 TL and >2000 TL. 

Both maternal and paternal levels of education were strikingly low in the present study. Good parental education may be expected to result in a better fed child [8]; however, some studies have determined there is no association between parental level of education and good child nutrition [9]. In the present study, there wasn’t a significant difference between mothers acting as primary caregivers according to the level of education.

There wasn’t a significant correlation in the present study between maternal employment status and the children’s eating behavior scores. According to the Turkey Demographic and Health Survey (TDHS) for 2013, 31% of women in Turkey work. In the present study, 53 (20.8%) of the mothers worked, which is below the Turkish national average [6]. Due to working outside the home, working mothers have problems feeding their children. Babies may refuse to feed when the mother starts working, which can be due to a change in the feeding schedule [10]. There wasn’t a significant correlation in the present study between mothers working and the duration of breastfeeding. We think that one of the reasons for this is that the number of days of maternity leave for working mothers in Turkey was increased and that they have also been given the right to take unpaid leave. Following six weeks of paid maternity leave after giving birth, milk leave of 1.5 h per day for six months was extended to 1.5 h per day for 13 months. If they wish, mothers also have the right to take 12 months of unpaid leave after the end of the paid maternity leave period [11]. Due to the low number of working mothers in the present study, accurate evaluation was not possible, and additional larger-scale research is needed for more definitive relevant conclusions.

Bottle feeding is widespread in Turkey and is known to result in such problems as decreased consumption of mother’s milk and obesity [12]. It was widely used (80.4%) in our study as well. Dental and hygiene problems have also been reported related to bottle feeding in several studies. The first 5 years are very important in child nutrition habits and oral hygiene, and parents should show an example for their children as role models [13]. 

A history of difficulty swallowing solid foods was present in 16.5% of the children in the study group. Difficulty swallowing solid foods is a frequently observed phenomenon during the transition to supplementary foods. Eating with a spoon during the transition to supplementary foods, chewing, self-feeding by holding foodstuffs with fingers, independently eating from a plate, and the ability to use a spoon and fork represent a child’s various feeding stages [14,15]. Although the present study group was aged 1-5 years, a high level of problems eating solid foods was observed.

Children aged 1-2 and children aged >2 years were evaluated separately in terms of self-feeding, the third of the eating behavior problems. As expected, children aged >2 years exhibited a greater ability to feed themselves, as compared to those aged <2-5 years (74.8% and 43.8%, respectively). Pre-school children become less obdurate and more obedient as they age, and they eat more comfortably in unfamiliar environments than younger children [6]. Terzi [15] reported that 53.9% of children ate with the assistance of another person, while 40.4% fed themselves. Kobak et al. [16] reported that children were able to self-feed from the age of 15 months and could eat unaided at age three years.

A high proportion (92.5%) of the present study group ate at the family dining table. Terzi [15] reported that only 17.3% of children in their study ate at the dining table. It is essential for a child aged >1 year to eat at the dining table; however, it should not be forgotten that a child will not be able to eat as fast as an adult, and patience is required [15,17].

A high rate of positivity (85.5%) was also determined in terms of regular means. Children aged 1-5 years express reactions to their families or surroundings by not eating; however, if a child exhibits normal growth and development if a child’s eating habits are not problematic, and if a child eats sufficiently for its requirements, then occasionally skipping a meal will not constitute a problem. Even if such irregularities persist for a few days, this will still not result in a major health problem [6,18].

Eating in 1-5-year-olds should last <30 min. Unfortunately, only 49.8% of children in the present study completed meals in <30 min. If a child quickly loses interest in eating and if eating lasts >1 h, then efforts must be made to reduce lack of attention to a minimum; however, there should be no insistence that portions be entirely consumed during meals. If a child is unwilling to eat, it will not do so. It is better to wait for some time and then suggest that the child continue to eat [6,19].

Compelling or intimidating a child into eating was noted in 22.4% of the present study group. According to the child feeding guideline published by the Turkish Ministry of Health in 2013, forcing or frightening a child into eating must be avoided [6,20]. The psychological conflict between an insistent mother and child is inevitable. Mothers frequently present to pediatricians because their children refuse to eat or because they vomit if they are forced to do so. A child should not be forced to eat by walking behind it with a plate and spoon. Many mothers make this mistake, particularly in the playing age [13]. A child should not be compelled to finish everything on its plate at every meal. Such behavior can lead to overeating or detestation of food. This in turn can subsequently result in obesity or eating problems [6,21].

In the present study, 50.3% of the children were encouraged to eat by means of television, games, or attention-distracting objects. Terzi [15] reported that 12.5% of children ate in front of the television. Allowing a child to watch television while eating or making use of some other device is not recommended. Care should be taken to ensure that stimulating objects that might distract a child’s attention, such as a television, should not be present [6]. In the present study, 22.4% of the children were compelled to eat by walking around behind them. Terzi [15] also reported that 5.8% of children were encouraged to eat by watching them from behind. Children must be taught from this age that eating is an essential activity. Encouraging the child to eat by singing, dancing, or following it with a plate, or exhibiting excessive haste or distraction behavior while feeding is not appropriate [6].

Keeping food in the mouth for extended periods of time is an indication of an eating behavior disorder in children and is suggestive of lack of appetite. This was reported by 50.3% of the mothers in the present study. On the other hand, fussy eating was noted in 54.5% of the present study’s children and is among the most difficult eating problems for caregivers of children aged 1-5 years. Frequently preparing the same food because the child likes it can eventually give rise to a lack of enthusiasm for that food. The most important factor is the attitude towards food exhibited by members of the family. A child will be influenced by the father, whom it regards as an authority figure in the family, the mother, to whom it feels proximity and who solves all its problems, and by siblings that it may be jealous of. Their forms of behavior predominate in terms of likes and reluctance [6].

In the present study, 60.8% of the children consumed snack foods. Snack food consumption can increase in the absence of regular meals. This is because children are less resistant to hunger than adults. When meals are not provided regularly a child may tend to snack more throughout the day and, therefore, be less hungry at mealtimes. For the same reason, frequent skipping of meals is not a good idea [6].

The mothers’ rating of their children having no or a moderate appetite, and having a good or very good appetite was correlated with the children’s eating behavior scores, which indicates that the questions about eating behavior were reliable and were accurately answered by the mothers. Eating problems in the family were most commonly reported in siblings, followed by fathers, and then mothers. Several studies of children with poor appetite have reported that their siblings generally have a poor appetite as well, which is largely attributed to genetics, family dynamics, and intrafamilial psychodynamics [17]; however, these factors were not investigated in the present study, which can be considered a limitation. Another limitation of the present study is the fact that due to its use of a questionnaire and cross-sectional design a cause and effect relationship could not be established. There are other certain limitations to our study as well. The most important of these is the lack of international reliability validity index studies of the survey questions asked. In similar studies to be carried out in the future, more participants can be used and questionnaires with reliability validity index.

The strengths of the study are that the questionnaire was administered face-to-face and that the consistency of the replies were compatible with other analyses. The mean eating behavior scores in the children with and without protein-energy malnutrition did not differ significantly, which also indicates the power of the questionnaire that was used.

## Conclusions

The present findings show there isn’t a significant association between eating behavior scores and such variables as type and time of delivery (vaginal or cesarean), maternal level of education, maternal employment status, maternal age, or the number of children in the family. Analysis of frequently observed negative eating behaviors showed that the bottle feeding rate was 80.4%, 38.8% of the children did not feed themselves, eating lasted >30 min in 50.2% of the children, and that children were encouraged to eat via compulsion or intimidation in 22.4% of the children. Problematic eating behavior was more common in the children with protein-energy malnutrition (based on WFH), as expected. In addition, the mean eating behavior score was higher in the children with mother-reported poor appetite than in those with mother-reported good appetite.

In conclusion, the eating behavior questions prepared for use in this research were compatible with the other findings, which shows that the questions were reliable and valid. Identification of eating problems in children aged 1-5 years and investigation of their causes are important for finding solutions. It is important to work with the family to identify solutions to problematic eating behavior and for pediatricians to follow-up children at regular intervals. 

The successful treatment of eating problems in children requires a multidisciplinary approach. In addition to a physician and psychologist, it is important that the treatment team include a dietician to monitor calorie intake and determine calorie targets, a speech therapist to examine eating-related motor and sensory problems, and a social services specialist to provide the requisite resources for the family.
